# Liquid Biopsy in Hepatobiliary and Pancreatic Malignancies: Current Evidence and Implications for Surgical Practice

**DOI:** 10.3390/cancers18203349

**Published:** 2026-10-16

**Authors:** Silvio Caringi, Antonella Delvecchio, Annachiara Casella, Valentina Ferraro, Matteo Stasi, Nunzio Tralli, Tommaso Maria Manzia, Riccardo Inchingolo, Michele Tedeschi, Riccardo Memeo

**Affiliations:** 1 Unit of Hepato-Biliary and Pancreatic Surgery, “F. Miulli” General Hospital, 70021 Acquaviva Delle Fonti, Italy; 2 Department of Medicine and Surgery, LUM University, 70010 Casamassima, Italy; 3 Transplant and HPB Unit, Department of Surgery Sciences, University of Rome Tor Vergata, 00133 Rome, Italy; 4 Unit of Interventional Radiology, F. Miulli Hospital, 70021 Acquaviva Delle Fonti, Italy

**Keywords:** liquid biopsy, hepatobiliary and pancreatic malignancies, circulating tumor DNA, surgical decision-making, molecular residual disease

## Abstract

Liquid biopsy is an emerging approach for the molecular characterization of hepatopancreatobiliary (HPB) malignancies through the analysis of circulating tumor DNA (ctDNA), circulating tumor cells (CTCs), and extracellular vesicles (EVs). These biomarkers may complement conventional imaging and serum markers by providing additional information on tumor biology and disease evolution. This narrative review examines the available evidence concerning the potential applications of liquid biopsy in HPB surgical practice, with particular emphasis on preoperative prognostic assessment, postoperative molecular residual disease detection, recurrence monitoring, and treatment adaptation. Among the different circulating biomarkers, ctDNA currently has the most developed perioperative evidence base. Studies involving pancreatic ductal adenocarcinoma, hepatocellular carcinoma, and biliary tract cancers have demonstrated associations between ctDNA detection and unfavorable oncological outcomes, particularly postoperative recurrence. Nevertheless, the available evidence remains predominantly observational, with considerable heterogeneity in patient populations, sampling protocols, analytical platforms, and outcome definitions. Although the integration of circulating biomarkers with conventional anatomical criteria has generated interest in the concept of “biological resectability”, this framework remains hypothetical and has not been prospectively validated for surgical treatment selection. Similarly, ctDNA-guided modifications of neoadjuvant therapy, adjuvant treatment, or postoperative surveillance have not yet demonstrated clinical benefit in HPB malignancies. Liquid biopsy should therefore currently be regarded as a promising investigational prognostic tool rather than an established determinant of surgical or oncological management. Standardized methodologies and prospective interventional trials are required before its routine clinical implementation.

## Simple Summary

Patients with hepatobiliary and pancreatic malignancies present a challenge for early diagnosis and carry a high recurrence rate even after potentially curative surgery. Liquid biopsy is a minimally invasive method of examination of tumor biomarkers circulating in the blood, which includes circulating tumor DNA, circulating tumor cells, and extracellular vesicles. These biomarkers can give additional information about the biology of tumors other than traditional radiologic and biochemical tests. In the course of surgical treatment, liquid biopsy can be used to assess the risk of recurrence prior to treatment and especially to detect minimal residual disease after surgery before its detection using imaging techniques. This review provides the currently available data on the use of liquid biopsy in pancreatic, liver, and biliary cancer, highlighting its possible significance for surgical practice. While promising results have been shown, currently available data support only its role in prognosis, but there is still insufficient data to use liquid biopsy alone for patient selection for surgery or changes in their therapy. Further prospective studies are needed before the introduction of liquid biopsy into clinical practice.

## 1. Introduction

Hepato-Pancreato-Biliary (HPB) cancers, including pancreatic ductal adenocarcinoma (PDAC), hepatocellular carcinoma (HCC), and biliary cancers, belong to the leading causes of death from cancer mainly due to their aggressive behavior and late diagnosis [1,2]. Despite substantial improvements in surgical treatment of patients with these diseases, the prognosis of such patients is hampered by the high risk of cancer recurrence, which occurs frequently within two years after surgery [3,4]. Early diagnosis and postoperative surveillance are one of the major needs in managing these patients.

In case of HPB cancers, conventional methods of diagnosis and surveillance are based on the application of cross-sectional imaging and blood biomarkers, such as carbohydrate antigen 19-9 (CA 19-9). These diagnostic approaches are considered limited in their sensitivity and specificity particularly for detecting early recurrent cancers and molecular residual disease [5,6]. Therefore, there is growing interest in the development of new and minimally invasive biomarkers to detect HPB cancers in real time [7,8].

Liquid biopsy was considered one of the most promising ways for conducting research, and there are numerous opportunities when liquid biopsy can be utilized, but it is yet to be seen whether liquid biopsy will be useful. This involves the use of different types of tumor-derived materials that may be found in biofluids, such as circulating tumor DNA (ctDNA), circulating tumor cells (CTCs), and extracellular vesicles including exosomes [9,10]. It is possible to utilize liquid biopsy as an attempt to solve the problem of tumor heterogeneity in both space and time due to the fact that it shows the molecular composition of the tumor.

Liquid biopsy is being investigated as a complementary tool for perioperative risk stratification and postoperative recurrence monitoring in HPB malignancies. Although circulating biomarkers may provide additional prognostic information beyond conventional anatomical and clinicopathological parameters, their ability to improve surgical patient selection or guide neoadjuvant treatment remains unproven. The integration of molecular biomarkers into the assessment of resectability should therefore be regarded as a research hypothesis rather than an established clinical decision-making strategy.

In this narrative review, we will discuss the current data on the utility of liquid biopsy in HPB cancers, with special attention to the perioperative period. The main novelty of our narrative review is the concept of “biological resectability” from the point of view of surgery: do the biomarkers in circulation allow for the detection of high-risk patients with undetected systemic disease or early recurrence? Since the available data are mainly prognostic but not treatment predictive, the suggested decision points remain hypotheses for future validation.

## 2. Literature Identification

This narrative review was developed through a focused examination of the literature indexed in PubMed/MEDLINE, complemented by the assessment of reference lists from relevant publications. The bibliographic coverage was defined as 1 January 2015 to 31 July 2026, with earlier landmark studies considered when directly relevant to the topic. 31 July 2026 was adopted as the publication cut-off for the evidence discussed in this review.

To enhance methodological transparency and facilitate reproducibility, a structured PubMed/MEDLINE search framework was developed using three complementary groups of terms: liquid biopsy technologies, hepatopancreatobiliary malignancies, and surgical or prognostic outcomes. Five Boolean combinations were formulated to address the overall HPB population and disease-specific evidence concerning pancreatic ductal adenocarcinoma, hepatocellular carcinoma, and biliary tract cancers. The complete search framework is presented in Table 1. These queries are provided as a reproducible search strategy rather than as a retrospectively reconstructed record of the original literature identification process; consequently, no historical search execution date or database retrieval counts are claimed.

Priority was given to original clinical investigations involving surgically treated or potentially resectable HPB malignancies, particularly studies reporting perioperative circulating biomarker assessment, postoperative recurrence, molecular residual disease, or survival outcomes. Both prospective and retrospective investigations were considered. Studies conducted exclusively in advanced or metastatic populations were included selectively when relevant to the biological rationale, molecular characterization, or systemic treatment implications of liquid biopsy, and their findings were distinguished from evidence directly applicable to surgical practice.

Study selection was guided by clinical relevance, methodological clarity, and the contribution of individual publications to the principal objectives of the review. Relevant consensus recommendations and selected clinical trial registrations were additionally considered to contextualize emerging applications and future research directions. As this work was conceived as a narrative review, no formal systematic screening process, predefined study-selection flow, risk-of-bias assessment, or meta-analysis was performed. Accordingly, the review provides a focused synthesis of representative evidence rather than an exhaustive systematic assessment of the literature.

Studies were selected for clinical relevance, methodological transparency, and their contribution to understanding potential applications in HPB surgery. Relevant professional recommendations and trial registrations were considered for context; this review does not claim an exhaustive registry or guideline search. In particular, no conclusions about the efficacy of ctDNA-guided treatment can be drawn from the existence of registered observational studies.

## 3. Types of Liquid Biopsy and Circulating Biomarkers

Liquid biopsy is an umbrella term referring to a diverse group of tumor products present in biological fluids, mostly peripheral blood. From those, the most researched in the field of HPB malignancies are ctDNA, CTCs, and extracellular vesicles (EVs), including exosomes. Each of those liquid biopsy products is uniquely important and significant, reflecting various features of tumor biology and development.

ctDNA is a collection of small DNA fragments released into the bloodstream by the means of apoptosis, necrosis, and secretion. In the case of HPB malignancies, ctDNA has demonstrated high potential as a liquid biopsy product for the identification of genetic mutations specific to tumors. In PDAC, mutations in ctDNA have been shown to be predominantly KRAS mutations associated with the size of the tumor, disease progression, and the risk of recurrence after surgery [11,12,13,14]. In HCC, mutations in ctDNA have been shown to involve TP53, CTNNB1 genes, and epigenetic abnormalities such as abnormal methylation [15,16,17]. In biliary tract cancer (BTC), profiling of ctDNA allows identification of potentially actionable genomic abnormalities. In late-stage diseases, profiling of the plasma has revealed IDH1 mutations, while FGFR2 fusion-positive cholangiocarcinoma acquires secondary FGFR2 mutations detectable via ctDNA during acquired resistance [18,19].

CTCs refer to intact malignant cells that detach from primary tumors or metastases to reach the bloodstream. Compared with ctDNA, CTCs allow for morphologic, phenotypic, and functional evaluation of single cells. CTCs have been linked to an unfavorable prognosis and metastatic nature in HPB cancers. The prognostic significance of CTCs needs to be considered based on specific tumor types. In patients with PDAC, pre-surgical phenotyping of CTCs has been linked to metastatic disease and overall survival, whereas in patients with HCC, EpCAM-positive CTCs have been related to worse post-operative survival rates [20,21]. These results are prognostic and assay-dependent but have not yet proven the clinical utility of CTC quantification.

Extracellular vesicles, particularly exosomes, are small vesicular bodies encapsulated in a membrane, which are actively secreted by tumor cells and carry proteins, lipids, DNA, and various RNAs, including microRNAs. They play roles in intercellular communication and modification of the tumor microenvironment. As far as biomarkers of HPB-related malignancies are concerned, tumor exosomes have been studied as potential diagnostic tools for HPB cancers. For instance, studies have focused on differentiating exosomal miRNAs and proteins. In one comparative analysis, increased expression of exosomal miR-10b, miR-21, miR-30c, and miR-181a in combination with decreased let-7a distinguished PDAC patients from chronic pancreatitis and healthy subjects; however, exosomal glypican-1 was not identified as a differential marker [22]. Contrarily, in the study on glypican-1, authors identified GPC1-positive exosomes as a potential biomarker for the early detection of pancreatic cancer [23]. In HCC, recent research has identified microRNAs delivered via extracellular vesicles as potential regulators of HCC cell proliferation; further claims concerning angiogenesis or immune escape need to be verified independently in HCC models [24].

Overall, all constituents of liquid biopsy have specific strengths and weaknesses, and their systematic evaluation might possibly increase the capacity for diagnosis and prognosis of HPB cancers. Systematic evaluation of different circulating biomarkers as a “multi-analyte” approach may be a way of overcoming the weaknesses of single liquid biopsies and capturing tumor heterogeneity and dynamics of tumor characteristics [7,25].

The principal circulating biomarkers and their potential surgical research applications are discussed above; quantitative data from selected perioperative studies are presented in Table 2.

Although ctDNA, CTCs, and EVs represent complementary components of liquid biopsy, their clinical development in the perioperative management of HPB malignancies is markedly different. Most available evidence addressing postoperative recurrence, molecular residual disease, and potential treatment adaptation derives from ctDNA-based investigations. In contrast, perioperative studies involving CTCs and EVs remain comparatively limited, with substantial variability in detection methods, biomarker definitions, and clinical endpoints. Consequently, the following sections predominantly focus on ctDNA, reflecting the current distribution of surgical evidence rather than an assumption of its biological superiority. Other emerging liquid biopsy approaches, including circulating cell-free RNA (cfRNA) and tumor-educated platelets (TEPs), are also being investigated as potential sources of molecular information. However, their clinical relevance for perioperative decision-making in HPB malignancies remains insufficiently established.

## 4. Clinical Applications of Liquid Biopsy in HPB Malignancies

Liquid biopsy for HPB cancers continues to progress in the realm of clinical application, and there is growing evidence of the potential clinical relevance of this approach. Liquid markers can be used for less invasive detection of changes in tumors and may play an important role in deciding on surgery approaches. Clinical applications of this technology are currently diverse and rather investigational.

### 4.1. Early Detection and Differential Diagnosis

One of the greatest challenges in HPB oncology is the timely diagnosis of cancer in its early stages, which is especially relevant in the case of PDAC and BTC, as these cancers are usually diagnosed at a late stage. The liquid biopsy technique has been considered a promising complementary tool to traditional diagnostics for the purpose of detection of tumor-related genetic abnormalities in asymptomatic or at-risk populations.

As far as PDAC is concerned, the use of the ctDNA technique in the detection of KRAS mutations has been shown to possess some promise in the context of early PDAC diagnosis; however, its diagnostic sensitivity is low owing to low concentrations of tumor DNA in blood when tumors are small/localized [32]. The use of ctDNA in combination with other tumor markers, such as protein and exosomal markers, has been shown to provide better sensitivity than any single marker alone [25,30,32,33]. Exosomal biomarkers need to be interpreted with regard to the specific analyte being assessed. Initially, glypican-1-positive exosomes were described as a biomarker candidate for pancreatic cancer [23], while another study involving exosomal microRNA discovered a multiple-miRNA signature discriminating between PDAC and chronic pancreatitis/healthy control cases [22].

In HCC, liquid biopsy may play a supplementary role alongside established surveillance approaches in high-risk populations. Plasma methylated-DNA panels have shown diagnostic potential, but these studies evaluate epigenetic signatures rather than general somatic-mutation detection [31]. In cholangiocarcinoma, plasma cfDNA concentration and targeted sequencing have been investigated as adjuncts to diagnosis. In a cohort of 62 patients with cholangiocarcinoma, 33 with benign biliary disease, and 30 healthy controls, cfDNA concentration discriminated cholangiocarcinoma from benign biliary disease with 82.3% sensitivity and 57.6% specificity at the reported threshold; these data support further study but do not establish liquid biopsy as a stand-alone test for indeterminate biliary strictures [34].

Overall, liquid biopsy remains investigational for early detection and screening in HPB malignancies.

Despite these encouraging results, however, the body of evidence consists of studies conducted on small samples, often of a retrospective nature or of a case–control design and carried out on a highly selected population. The sensitivity of the liquid biopsy in cases of early-stage cancer is poor, especially where tumors have a low amount of ctDNA. Moreover, there is much variability in the methods adopted, with no standard criteria, thus making comparison difficult. The role of liquid biopsy in the early diagnosis of cancer remains experimental.

### 4.2. Prognostic Stratification and Preoperative Assessment

Proper risk stratification prior to surgery is vital in order to ensure optimal selection of candidates who will benefit from surgical resection. As the assessment of tumor biology is now feasible in addition to standard anatomical parameters, the possibility of early recurrence in cases where there is a high risk of recurrence but technically resectable tumors cannot be ruled out. From the surgical perspective, these results raise a number of questions about whether anatomical resectability alone would constitute sufficient criteria for undergoing upfront surgery. “Biological resectability” is the concept that was recently introduced in this context and includes ctDNA.

However, in PDAC, preoperative ctDNA indicates unfavorable tumor biology and not occult metastasis. A prospective biomarker study on localized PDAC showed that the presence of KRAS mutation in ctDNA before surgery was seen in 62% (23/37) of patients and was related to poor recurrence-free survival (RFS) and overall survival (OS) with a hazard ratio of 4.1 [27]. The presence of ctDNA has been reported to be related to increased recurrence rate in another cohort of resected PDACs [28]. Thus, the presence of ctDNA indicates increased systemic relapse risk, but it does not imply the presence of radiologically occult metastasis. In PDAC, CTC positivity has also been linked to poor clinicopathological characteristics and survival rates using different platforms and thresholds [20].

In HCC, preoperative ctDNA has been found to correlate with tumor burden, microvascular invasion, and post-hepatectomy recurrence. In a study in 81 patients undergoing surgery, preoperative ctDNA was detected in 70.4% of patients and independently correlated with recurrence postoperatively, while the mutation allele frequency postoperatively correlated with recurrence as well [35]. This information provides data for prognostic enrichment and not for the selection of surgery versus non-surgical treatment.

Regarding BTC, genomic ctDNA data have been gathered primarily in the setting of advanced/metastatic disease. In a 124-patient cohort of advanced BTC, plasma ctDNA analysis identified actionable genomic alterations, but the study did not include anatomically resectable disease or the use of perioperative information [19]. It should be noted that this information cannot be directly extrapolated to surgical decision-making.

Evidence supporting the preoperative application of ctDNA in patients with resectable BTC remains substantially less developed than evidence derived from advanced-stage populations. Available perioperative investigations have primarily focused on postoperative recurrence prediction, while studies specifically validating preoperative ctDNA detection for surgical risk stratification remain limited. Furthermore, differences in anatomical subtypes, tumor burden, biliary obstruction, and assay sensitivity complicate the interpretation and generalizability of circulating biomarker results. Consequently, preoperative ctDNA testing cannot currently be recommended as an independent criterion for determining resectability or selecting neoadjuvant treatment in BTC.

Accordingly, preoperative liquid biopsy should currently be interpreted as a prognostic adjunct rather than a validated treatment-selection test.

## 5. Surgical Decision-Making and Biological Resectability

In the management of HPB malignancies, anatomy-based criteria have been utilized for making decisions about the surgery. However, this approach does not fully account for tumor biology that is another significant aspect in terms of deciding on the prognosis of cancer. Liquid biopsy presents an opportunity for addressing the issue of tumor biology in the management of resectability.

For pancreatic cancer, detectable ctDNA prior to surgery is a consistent marker of increased risk of recurrence and poor outcomes in patients with localized or resectable disease [26,27,28,36]. The association generates a testable hypothesis according to which the treatment or neoadjuvant therapy would benefit those patients that are ctDNA positive, but there is no proof at the moment that ctDNA can indicate a greater benefit from neoadjuvant therapy than from upfront surgery. On the other hand, the absence of ctDNA does not mean that the patient has favorable biology and thus should undergo surgery right away.

For HCC, biomarkers circulating in the bloodstream could provide further information in terms of recurrence risk stratification prior to resection or liver transplant surgery. However, validated cut-offs for the choice between the two modalities remain to be defined [17,21,35]. For BTC, data on advanced-stage disease cannot yet be utilized to make decisions on whether surgery should be performed in an anatomically operable patient cohort [19].

None of the above decision points is currently supported by a validated ctDNA threshold or a prospective study showing benefits of ctDNA-driven approach on patient outcome. Therefore, it is best viewed as a hypothesis-generating scheme.

### 5.1. Postoperative Monitoring and Detection of Molecular Residual Disease

Some of the most promising areas where liquid biopsy can be implemented in HPB surgery include identification of molecular residual disease (MRD) following curative surgical resection of various HPB tumors. Conventional imaging techniques cannot reveal residual disease on a microscopic scale, hence the delay in diagnosis of recurrence. Residual disease at the molecular level could be detected using ctDNA analysis. This holds some implications for post-surgery care for surgeons. Postoperative ctDNA detection is associated with an increased risk of recurrence and may indicate molecular residual disease. However, ctDNA positivity does not definitively establish the presence or anatomical location of residual malignancy. Furthermore, current evidence does not demonstrate that modifying surveillance schedules or initiating, intensifying, or withholding adjuvant treatment solely on the basis of ctDNA results improves patient outcomes.

In PDAC, the presence of postoperative ctDNA is one of the most reproducible signals from the liquid biopsy for predicting outcomes in the surgical literature. In a small cohort of 35 patients, Lee et al. found KRAS mutant ctDNA in 37% of cases (13/35); all 13 ctDNA-positive patients subsequently developed recurrence, with postoperative positivity associated with poor RFS (HR 5.4) and OS (HR 4.0) [27]. Similarly, Groot et al. examined postoperative ctDNA in PDAC surgery and found strong correlations with recurrence [28]. These data support postoperative risk stratification, although the low sensitivity of a single negative test cannot be used to rule out residual disease.

In HCC, postoperative ctDNA has been shown to predict recurrence following potentially curative surgery. In a cohort of 96 patients, recurrence was found in 60.9% of ctDNA-positive patients compared to 27.8% of ctDNA-negative patients, and postoperative ctDNA was an independent predictor of disease-free survival (DFS) (HR 6.074; 95% CI 2.648–13.929) and OS (HR 4.829; 95% CI 1.508–15.466) [37]. In another study with 41 prospectively enrolled patients, ctDNA was identified in 46% of patients following surgery, and the serial postoperative positivity was correlated with poor RFS [29].

The postoperative experience with BTC is already accumulating through studies on surgically treated populations. Thus, in a multi-center study of 56 patients with stages I-III BTC undergoing surgery, ctDNA detection within the molecular residual disease window was associated with significantly shortened RFS (HR 26; 95% CI 2.6–265), and the same was true regarding detection during surveillance (HR 20; 95% CI 2.6–153) [38]. The earlier study by Ettrich et al. [39] focused on diagnostic/prognostic genotyping in cholangiocarcinoma and cannot be cited as evidence of postoperative detection of residual disease.

Postoperative ctDNA, thus, can be interpreted rather as a marker of a risk of recurrence and possible molecular residual disease than as definitive evidence of occult metastasis or as a sole rationale for changing treatment approach.

### 5.2. Guiding Adjuvant and Systemic Therapies

Additionally, liquid biopsy may find use in future monitoring of adjuvant and systemic therapies. It is worth noting that positive ctDNA status after surgery is presently a predictor of recurrence due to its high risk; it has never been confirmed as a predictor of patients benefiting from adjuvant therapies or as an exclusion criterion for these therapies. False negatives should be taken into account when considering early-stage HPB cancers due to low tumor shedding.

Serial ctDNA testing during adjuvant therapy is under investigation in PDAC as a dynamic marker. Recurrence has been seen in all 13 patients with postoperative ctDNA in the prospective localized-PDAC study by Lee et al. and in seven who had gemcitabine-based adjuvant chemotherapy [27]. Newer postoperative series have reported that ctDNA presence during the adjuvant/surveillance period correlates with relapse; however, prospective interventional studies need to be conducted before any change in treatment (intensification, modification, or omission) of therapy on the basis of ctDNA kinetics [40]. In HCC, ctDNA is able to identify tumor-specific genomic changes, which might correlate with recurrence and treatment efficacy. However, no validated ctDNA level is available for selecting targeted and immunotherapies [29,35,37].

In advanced BTC, ctDNA profiling is able to detect alterations such as IDH1 mutations [19], and sequential ctDNA analysis has shown secondary FGFR2 mutations which are linked to acquired resistance to FGFR blockade in FGFR2 fusion-positive cholangiocarcinoma [18]. This is an application of liquid biopsy in advanced disease, distinct from perioperative management.

Although prospective observational studies have strengthened the evidence supporting ctDNA as a prognostic biomarker in resectable HPB malignancies, an important distinction must be made between prospective biomarker validation and interventional trials in which treatment decisions are guided by ctDNA results. Most available studies demonstrate associations with recurrence and survival rather than improved outcomes following biomarker-guided interventions. Future trials should evaluate whether ctDNA-guided treatment intensification, adjuvant therapy modification, or surveillance adaptation improves clinical outcomes compared with conventional management. Until such evidence becomes available, ctDNA should be considered complementary prognostic information rather than an independent criterion for treatment allocation.

The clinical role of liquid biopsy in treatment selection is unknown. Randomized controlled trials are required to determine whether the use of ctDNA to alter management affects survival.

Recent observational data in surgically treated PDAC reinforce the prognostic association of perioperative and postoperative ctDNA detection with recurrence, but do not demonstrate improved outcomes from ctDNA-guided treatment changes. The ESMO Precision Medicine Working Group has advised against routine MRD-directed treatment decisions in early-stage cancer outside research settings, while the National Cancer Institute GI Oncology ctDNA Working Group has emphasized the need for rigorous prospective trials and validated endpoints [41,42]. These recommendations are cross-cancer methodological context rather than HPB-specific proof of clinical utility.

## 6. Quantitative Evidence from Representative Perioperative Studies

Table 2 summarizes selected original studies evaluating the prognostic significance of perioperative ctDNA assessment in patients with potentially resectable HPB malignancies. Studies were selected for their direct relevance to surgical practice, availability of clearly defined patient populations, description of biomarker assessment and sampling timing, and reporting of clinically meaningful outcomes, particularly recurrence-free and overall survival. Both prospective and retrospective investigations were considered. Studies exclusively involving unresectable or metastatic disease were excluded from this perioperative evidence table. The table is intended to provide a representative narrative overview rather than an exhaustive or systematically selected evidence synthesis. Importantly, the studies predominantly demonstrate prognostic associations and do not establish the clinical utility of ctDNA-guided treatment decisions.

## 7. Limitations and Clinical Implementation

A number of barriers currently hinder the routine use of liquid biopsy in HPB surgery pathways. First, the research is mostly based on retrospective cohorts and small prospective biomarker studies featuring diverse tumor stages, sampling intervals, endpoints, and methods used for analysis. Second, pre-analytical factors such as collection tubes, processing time, amount of plasma collected, storage conditions, and analytical parameters such as tumor-informed or tumor-naïve assays, ddPCR versus NGS, panels of genes tested, variant thresholds, and adjustment for clonal hematopoiesis can significantly influence results [9,10]. Clonal hematopoiesis may introduce non-tumor variants into plasma sequencing results; matched white blood cell sequencing can help distinguish these from tumor-derived alterations [9]. Third, shedding of ctDNA is often low in localized HPB tumors, which means that a negative test result cannot exclude the presence of microscopic residual or metastatic disease; hence, false negatives are a serious issue with regard to possible de-escalation of surgery, surveillance, and adjuvant therapy. Fourth, no consensus exists on threshold positivity and the clinically meaningful changes in ctDNA dynamics. Finally, the critical missing link in the evidence is the clinical usefulness of biomarkers: almost all studies provide evidence about the prognostic power of ctDNA but none prove that treatment guided by ctDNA improves patient outcomes prospectively.

To improve reproducibility and facilitate comparisons across studies, future investigations should adopt standardized pre-analytical and analytical protocols [9,10]. Blood collection procedures should specify the type of collection tube, including whether conventional EDTA tubes or specialized cell-stabilizing tubes were used, together with the interval between blood collection and plasma separation. Plasma processing should follow validated procedures designed to minimize leukocyte lysis and contamination by non-tumor-derived DNA. Investigators should report the initial blood volume, recovered plasma volume, centrifugation conditions, storage temperature, duration of storage, and number of freeze–thaw cycles. Analytical reporting should include the assay platform, sequencing depth or analytical sensitivity, limit of detection, variant allele frequency thresholds, quality-control procedures, and methods used to distinguish tumor-derived alterations from clonal hematopoiesis. Whenever feasible, paired leukocyte sequencing should be considered to reduce false-positive interpretations. Finally, the definition of ctDNA positivity, the timing of perioperative sampling, and the criteria used to establish molecular residual disease should be explicitly reported and prospectively validated. These measures would improve analytical comparability but would not, by themselves, establish clinical utility. Taken together, these limitations underscore that liquid biopsy should currently be considered a complementary investigational tool rather than a standard component of clinical decision-making in HPB malignancies.

## 8. Future Directions

Future research in liquid biopsy for HPB malignancies should prioritize the transition from prognostic biomarker validation to the demonstration of clinical utility. Although ctDNA has shown promising associations with recurrence and survival, its ability to improve patient outcomes through biomarker-guided therapeutic interventions remains insufficiently established. The recommendations of the European Society for Medical Oncology (ESMO) Precision Medicine Working Group and the National Cancer Institute Gastrointestinal Oncology ctDNA Working Group emphasize the need for prospective interventional validation, standardized methodologies, and clinically meaningful endpoints before MRD testing can be incorporated into routine treatment decisions [41,42].

In PDAC, the DYNAMIC-Pancreas study (ACTRN12618000335291) represents an important step toward evaluating ctDNA-informed adjuvant treatment. The registered study includes a randomized comparison of standard versus ctDNA-guided postoperative management among patients previously treated with neoadjuvant chemotherapy, together with a separate cohort undergoing upfront surgery. Preliminary results from the upfront-surgery cohort, presented at the 2024 American Society of Clinical Oncology Annual Meeting, included 102 patients and demonstrated the feasibility of postoperative ctDNA-informed adjuvant treatment allocation. Postoperative ctDNA was detected in approximately 40% of patients, and ctDNA-positive patients experienced shorter median recurrence-free survival than ctDNA-negative patients (13 versus 22 months). However, these findings primarily support feasibility and prognostic stratification rather than demonstrating improved survival through ctDNA-guided treatment modification [43,44].

Other prospective studies are investigating the role of ctDNA kinetics during neoadjuvant treatment. The observational study NCT04616131 was designed to evaluate ctDNA detection and clearance in patients receiving chemotherapy for non-metastatic pancreatic cancer, examining associations with radiological response, pathological regression, and conventional tumor markers. Such investigations may help clarify whether longitudinal molecular monitoring provides information beyond anatomical imaging and serum carbohydrate antigen 19-9. Nevertheless, the ability of ctDNA clearance to guide surgical timing, determine the duration of neoadjuvant therapy, or identify patients who would benefit from alternative systemic treatment remains unvalidated [45].

In BTC, prospective investigations are similarly exploring the potential of postoperative ctDNA monitoring to personalize adjuvant treatment. The interventional study NCT06171321 evaluates a ctDNA-guided treatment strategy following radical surgery. Preliminary findings presented in 2025 described 38 patients undergoing serial molecular monitoring at predefined perioperative and surveillance time points. Persistent or newly detectable ctDNA prompted treatment escalation in selected patients, while ctDNA positivity during adjuvant therapy was associated with shorter disease-free survival. However, the small sample size, limited follow-up, and preliminary nature of these findings prevent conclusions regarding the survival benefit of ctDNA-guided treatment escalation [46].

For HCC, future investigations should focus on the prospective validation of circulating biomarkers for recurrence-risk stratification following hepatic resection and liver transplantation. Serial ctDNA assessment may potentially complement established clinicopathological predictors and conventional imaging, particularly in patients at increased risk of early recurrence. However, important uncertainties remain regarding the optimal timing of sampling, the interpretation of postoperative molecular positivity, and the ability of ctDNA-guided surveillance or adjuvant treatment to improve long-term outcomes. Prospective studies should therefore distinguish earlier molecular detection from demonstrable clinical benefit and carefully evaluate the consequences of false-positive and false-negative findings.

Beyond ctDNA alone, multi-analyte approaches combining circulating tumor cells, extracellular vesicles, circulating RNA, epigenetic signatures, circulating proteins, and conventional clinical parameters may improve the characterization of tumor heterogeneity and disease evolution. Existing multi-biomarker research provides a rationale for integrating complementary molecular signals, although evidence demonstrating incremental clinical benefit in perioperative HPB populations remains limited [7,25,32]. Future investigations should incorporate standardized blood collection and processing protocols, validated analytical platforms, reproducible positivity thresholds, and predefined sampling intervals to facilitate comparisons between studies and ensure clinical applicability.

From a surgical perspective, the ultimate objective should be the development of prospectively validated multidisciplinary decision-making models integrating anatomical resectability, clinicopathological characteristics, treatment response, and molecular biomarkers. Such approaches may eventually help identify patients who could benefit from intensified systemic treatment, optimize postoperative surveillance, and refine treatment sequencing. Nevertheless, ctDNA positivity should not independently justify withholding potentially curative surgery, while ctDNA negativity should not be interpreted as evidence of favorable tumor biology or absence of microscopic disease.

Future interventional trials should prioritize clinically meaningful endpoints, including recurrence-free survival, overall survival, quality of life, and cost-effectiveness, rather than relying exclusively on molecular detection or clearance. Until prospective evidence demonstrates improved patient outcomes, liquid biopsy should remain a complementary investigational tool within multidisciplinary HPB oncology rather than an independent determinant of surgical or oncological management.

## 9. Conclusions

Liquid biopsy represents a promising approach for improving the molecular characterization and prognostic assessment of HPB malignancies. Among the available circulating biomarkers, ctDNA currently provides the most substantial perioperative evidence, particularly regarding postoperative recurrence-risk stratification and the detection of possible molecular residual disease. Evidence supporting the surgical applications of CTCs and extracellular vesicles remains comparatively limited.

Despite these encouraging findings, the available literature is predominantly observational and characterized by substantial methodological heterogeneity. Importantly, the prognostic value of ctDNA should not be confused with demonstrated clinical utility. Neither preoperative ctDNA positivity nor postoperative molecular detection currently provides sufficient evidence to independently determine surgical eligibility, select neoadjuvant or adjuvant treatments, or modify established surveillance protocols.

The concept of biological resectability, integrating molecular and anatomical tumor characteristics, remains an attractive but unvalidated framework. Future investigations should prioritize standardized sampling and analytical methodologies, prospective biomarker validation, and interventional trials assessing whether ctDNA-guided management improves survival, quality of life, and cost-effectiveness. Until such evidence becomes available, liquid biopsy should be considered a complementary investigational tool within multidisciplinary HPB oncology rather than a replacement for established clinical decision-making criteria.

## Figures and Tables

**Table 1:** Reproducible PubMed/MEDLINE search framework for literature identification. The strategy comprises three Boolean concept blocks (A–C) and five broad or disease-specific combinations (Q1–Q5). Each query can be reproduced by substituting the complete search syntax for the corresponding concept blocks and applying the publication-date restriction 2015/01/01:2026/07/31[dp]. The framework documents the search strategy used to structure the narrative review; it does not represent a verified historical search log or imply systematic study selection.

Block/Query	PubMed Syntax/Combination
A—Biomarker terms	(“liquid biopsy”[tiab] OR “circulating tumor DNA”[tiab] OR “circulating tumour DNA”[tiab] OR ctDNA[tiab] OR cfDNA[tiab] OR “circulating tumor cells”[tiab] OR CTCs[tiab] OR “extracellular vesicles”[tiab] OR exosomes[tiab])
B—HPB malignancies	(“pancreatic cancer”[tiab] OR “pancreatic ductal adenocarcinoma”[tiab] OR PDAC[tiab] OR “hepatocellular carcinoma”[tiab] OR HCC[tiab] OR cholangiocarcinoma[tiab] OR “biliary tract cancer”[tiab] OR “biliary tract cancers”[tiab])
C—Surgical/prognostic terms	(surg*[tiab] OR resect*[tiab] OR perioperative[tiab] OR postoperative[tiab] OR “molecular residual disease”[tiab] OR “minimal residual disease”[tiab] OR recurren*[tiab] OR surveillan*[tiab] OR prognos*[tiab])
Q1—Broad HPB	A AND B
Q2—Perioperative HPB	A AND B AND C
Q3—PDAC perioperative	A AND (“pancreatic cancer”[tiab] OR “pancreatic ductal adenocarcinoma”[tiab] OR PDAC[tiab]) AND C
Q4—HCC perioperative	A AND (“hepatocellular carcinoma”[tiab] OR HCC[tiab]) AND C
Q5—BTC perioperative	A AND (cholangiocarcinoma[tiab] OR “biliary tract cancer”[tiab] OR “biliary tract cancers”[tiab]) AND C

**Table 2:** Representative quantitative evidence for liquid biopsy in surgically relevant HPB settings. MRD, molecular residual disease; MAF, mutant allele frequency; RFS, recurrence-free survival; OS, overall survival; NGS, next-generation sequencing; ddPCR, droplet digital PCR; BTC, biliary tract cancer; DFS, disease-free survival.

Study	Tumor/Design	N	Analyte/Platform	Sampling	Key Quantitative Result	Evidence for Surgical Use
Lee et al., 2019 [26]	Localized PDAC; prospective biomarker study	112 enrolled; 81 resected	KRAS-mutant ctDNA; SafeSeqS	Preoperative and postoperative	Preoperative detection: 62% (23/37), RFS HR 4.1; Postoperative detection: 37% (13/35), RFS HR 5.4, OS HR 4.0; Recurrence in all 13 postoperative ctDNA-positive patients	Prognostic stratification; no demonstrated benefit from ctDNA-guided treatment
Groot et al., 2019 [27]	Resected PDAC; clinical cohort	59	KRAS mutation-specific ctDNA; ddPCR	Perioperative and surveillance	Sensitivity 90%, specificity 88% for recurrence detection; median lead time of 84 days compared with conventional detection	Potential postoperative MRD and recurrence monitoring; clinical utility unproven
Wang et al., 2020 [28]	HCC following curative hepatectomy	81	Mutation-specific ctDNA; ddPCR	Preoperative and postoperative	Preoperative ctDNA detection: 70.4% (57/81); ctDNA positivity independently associated with recurrence; postoperative mutant allele frequency changes correlated with recurrence	Prognostic stratification; no validated treatment-selection threshold
Ye et al., 2022 [29]	Early/intermediate-stage HCC following resection	96 enrolled; 95 classified by postoperative ctDNA status	ctDNA; hybrid-capture NGS	Postoperative	Recurrence: 60.9% (14/23) in ctDNA-positive versus 27.8% (20/72) in ctDNA-negative patients; DFS HR 6.074 (95% CI 2.648–13.929); OS HR 4.829 (95% CI 1.508–15.466)	Postoperative recurrence-risk stratification
Zhu et al., 2022 [30]	HCC; prospective longitudinal cohort	41	ctDNA; NGS	Preoperative and serial postoperative	Preoperative detection: 63.4%; postoperative detection: 46%; serial positivity associated with shorter RFS	Dynamic prognostic monitoring
Yu et al., 2025 [31]	Stage I–III BTC following curative resection; multicenter retrospective cohort	56	Tumor-informed ctDNA assay	Postoperative MRD window and surveillance	MRD-window positivity: RFS HR 26 (95% CI 2.6–265); surveillance positivity: RFS HR 20 (95% CI 2.6–153)	Strong prognostic association; benefit of ctDNA-guided interventions unproven

## Data Availability

No new data were created or analyzed in this study. Data sharing is not applicable to this article.

## Abbreviations

AFP	Alpha-fetoprotein
BTC	Biliary Tract Cancer
CA 19-9	Carbohydrate antigen 19-9
CTCs	Circulating tumor cells
ctDNA	Circulating Tumor DNA
DFS	Disease-free survival
HCC	Hepatocellular carcinoma
HPB	Hepato-Pancreato-Biliary
MRD	Molecular Residual Disease
OS	Overall survival
PDAC	Pancreatic Ductal Adenocarcinoma
RFS	Recurrence-free survival
